# Nucleic Acid-Based Treatments Against COVID-19: Potential Efficacy of Aptamers and siRNAs

**DOI:** 10.3389/fmicb.2021.758948

**Published:** 2021-11-08

**Authors:** Javad Khanali, Mohammadreza Azangou-Khyavy, Yasaman Asaadi, Monire Jamalkhah, Jafar Kiani

**Affiliations:** ^1^School of Medicine, Shahid Beheshti University of Medical Sciences, Tehran, Iran; ^2^Department of Biotechnology, College of Science, University of Tehran, Tehran, Iran; ^3^Department of Biochemistry, Microbiology and Immunology, University of Ottawa, Ottawa, ON, Canada; ^4^Ottawa Hospital Research Institute, Ottawa, ON, Canada; ^5^Department of Molecular Medicine, School of Advanced Technologies in Medicine, Iran University of Medical Sciences, Tehran, Iran

**Keywords:** aptamer, COVID-19, RNA interference, aptamer–siRNA chimera, siRNA, virus neutralization

## Abstract

Despite significant efforts, there are currently no approved treatments for COVID-19. However, biotechnological approaches appear to be promising in the treatment of the disease. Accordingly, nucleic acid-based treatments including aptamers and siRNAs are candidates that might be effective in COVID-19 treatment. Aptamers can hamper entry and replication stages of the SARS-CoV-2 infection, while siRNAs can cleave the viral genomic and subgenomic RNAs to inhibit the viral life cycle and reduce viral loads. As a conjugated molecule, aptamer–siRNA chimeras have proven to be dual-functioning antiviral therapy, acting both as virus-neutralizing and replication-interfering agents as well as being a siRNA targeted delivery approach. Previous successful applications of these compounds against various stages of the pathogenesis of diseases and viral infections, besides their advantages over other alternatives, might provide sufficient rationale for the application of these nucleic acid-based drugs against the SARS-CoV-2. However, none of them are devoid of limitations. Here, the literature was reviewed to assess the plausibility of using aptamers, siRNAs, and aptamer–siRNA chimeras against the SARS-CoV-2 based on their previously established effectiveness, and discussing challenges lie in applying these molecules.

## Introduction

As of March 30, 2021, the COVID-19 resulted in over 120,000,000 confirmed cases and 2,500,000 confirmed deaths globally (WHO, 202AD). Although the rising number of vaccines are being introduced and approved (FDA News Release, 2020; Zhao et al., 2020; Jones and Roy, 2021; U.S. FOOD & DRUG ADMINISTRATION, 2021), based on the pandemic’s magnitude, there is an urgent need to develop safe and effective therapies as well.

Accordingly, various therapeutic approaches targeting different aspects of Severe Acute Respiratory Syndrome Coronavirus 2 (SARS-CoV-2) pathogenesis have been proposed since the beginning of the pandemic. These approaches range from dampening SARS-CoV-2 entrance host cells to modulating host immune responses (Li et al., 2020a). Despite significant efforts, there are currently no approved treatments for COVID-19 (Tu et al., 2020). However, biotechnological approaches appear to be promising in treating the COVID-19 (Abbott et al., 2020; Jiang et al., 2020; Nguyen et al., 2020).

In this regard, nucleic acid-based molecules, which had been effective against the SARS-CoV, are also being considered as potentially effective drugs against the SARS-CoV-2 (Piyush et al., 2020). These nucleic acid therapeutics which include but are not limited to antisense oligonucleotides, siRNA, RNA-targeting clustered regularly interspaced short palindromic repeats/CRISPR-associated protein (CRISPR-Cas), vaccines, aptamers, and ribozymes are reviewed elsewhere as anti-COVID drugs (Le et al., 2020; Piyush et al., 2020; Uludağ et al., 2020). But the focus of this review is aptamers and small interfering RNAs (siRNAs) as two potential candidates that might be effective in the treatment of the COVID-19. Here, we first provide a nucleic acid-based perspective on the treatment of the COVID-19 by reviewing the studies conducted to elucidate aptamers and siRNAs efficacy; then, an alternative antiviral approach adopting aptamer–siRNA chimeras (AsiCs) is presented that benefits from both aptamers and siRNAs-mediated antiviral effects. Finally, the challenges that need to be overcome in implementing these treatments are discussed.

## Aptamers Against the SARS-CoV-2

Aptamers are simple genetic entities composed of short nucleic acid sequences that can specifically bind to various molecules (Kinghorn et al., 2017). These molecules are isolated through the Systematic Evolution of Ligands by Exponential Enrichment (SELEX) procedure. In this procedure, a random library of constructed aptamers is exposed to the target molecule, and after washing out the unbound aptamers, the selected aptamers are amplified (Ellington and Szostak, 1990; Tuerk and Gold, 1990).

Based on the target molecule function, aptamers can be used as therapeutic molecules. For example, vascular endothelial growth factor can be targeted by aptamers to treat age-related macular degeneration (AMD) and other ocular disorders, some of which have reached clinical phases (Martin et al., 2002; Guyer et al., 2003; Gragoudas et al., 2004; Drolet et al., 2016). Among these anti-VEGF aptamers, *Macugen* is approved for safe and effective use in AMD (Drolet et al., 2016). Furthermore, aptamers have been utilized as anti-coagulative agents through antagonizing coagulation factor IXa, thrombin, and von Willebrand factor (Nimjee et al., 2017). Besides, in the field of cancer-targeted therapy, aptamers have been propitious. Accordingly, aptamers against nucleolin have shown anti-proliferative effects on different cancers (Nimjee et al., 2017) and have also managed to enter clinical trials (Rosenberg et al., 2014). Aptamers against PSMA (Lupold et al., 2002; Dassie et al., 2014), ErbB-2 (Mahlknecht et al., 2013), AXL (Cerchia et al., 2012), MUC1 (Ferreira et al., 2009), CEA (Correa et al., 2014), and CTLA4 (Gregor et al., 2004) have also been investigated as cancer treatment strategies.

Aptamers are potentially effective treatments against viral infections as well. They can disrupt the attachment and replication of viruses by binding to and blocking different molecules (Zou et al., 2019). Initially, the first antiviral aptamer was used against HIV. In the study conducted by Sullenger et al., the RNA aptamer was used to bind to the Tat and cyclin T1 proteins in CD4^+^ T cells, which prevented viral replication (Sullenger et al., 1991). Furthermore, aptamers can inhibit various other stages of HIV infection by binding to targets such as reverse transcriptase and integrase (Ditzler et al., 2011; Labib et al., 2012; Magbanua et al., 2013). Influenza viruses are other viral pathogens successfully targeted by aptamers. The HA surface glycoprotein of the influenza virus, which binds to sialic acid receptors on the host cell surface, has been targeted by different aptamers to disrupt the virus entry (Cheng et al., 2008; Musafia et al., 2014). Another stage of influenza virus infection is the transcription that can also be targeted by aptamers against the virus endonucleases (Yuan et al., 2015). Similarly, the glycoprotein D of the HSV-1 can be targeted by both DNA and RNA aptamers to inhibit the virus entry to host cells (Gopinath et al., 2012; Yadavalli et al., 2017). It is also worth noting that the HBV core protein and HCV NS5B protein could be targeted by aptamers to interfere with the extracellular DNA synthesis and virus replication, respectively (Zhang et al., 2009; Lee et al., 2013). Moreover, in the case of SARS-CoV and SARS-CoV-2, aptamer-based biosensors have shown efficacy in diagnostic approaches (Cho et al., 2011; Roh and Jo, 2011; Samson et al., 2020).

**Figure 1:**
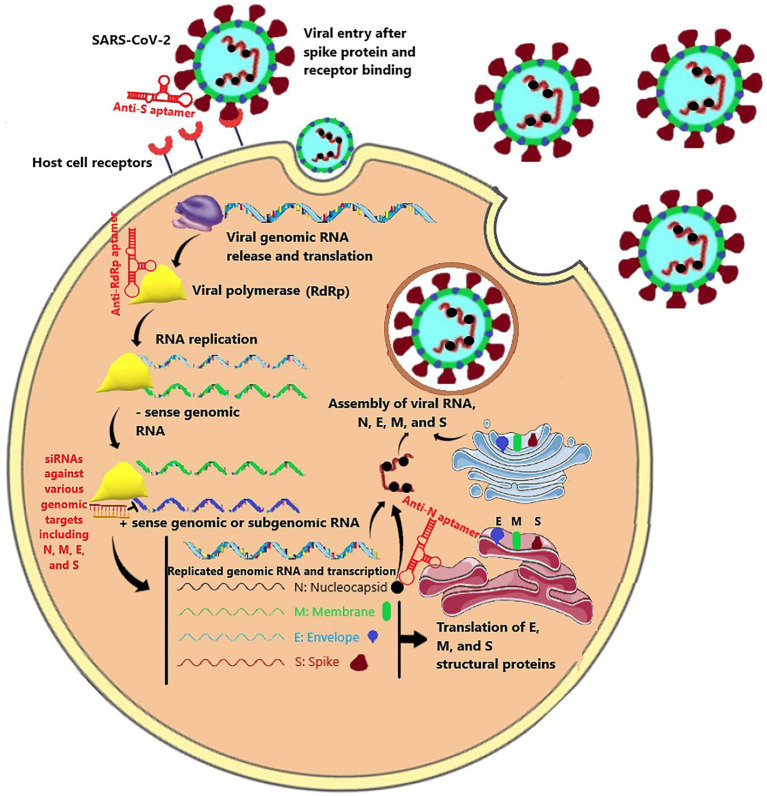
The SARS-CoV-2 life cycle consisted of virus entry mediated by the spike protein, viral RNA replication mediated by RdRp polymerase, transcription and translation of nucleocapsid (N), envelope (E), membrane (M), and spike (S), and new virus assembly. Aptamers and siRNA could be directed against various stages of this life cycle.

According to aptamers’ antiviral effects, using them as a therapeutic agent in the fight against the COVID-19 is not unimaginable (Torabi et al., 2020). Accordingly, the entry and replication stages of the SARS-CoV-2 infection process could be hampered by aptamers (Figure 1). These two objectives could be accomplished by targeting several intractable sequences in the receptor-binding domain (RBD) of the spike protein and RNA-dependent RNA polymerase (RdRp; Weisshoff et al., 2020). For example, the aptamer BC 007, which is in phase 2 clinical trial for congestive heart failure (The Persistence of Autoantibody Neutralisation by BC 007 in Patients With Chronic HFrEF and Autoantibodies Against the Beta1-Adrenergic Receptor - Full Text View - ClinicalTrials.gov), has been shown to be efficient in targeting the RBD of spike protein and the RdRp of the SARS-CoV-2 (Weisshoff et al., 2020). Furthermore, Song et al. suggested aptamer sequences against RBD of SARS-CoV-2 (Song et al., 2020). The researchers conducted SELEX on the artificially expressed RBDs on microspheres (Sun et al., 2021). They discovered and optimized two aptamer sequences with high binding affinities being able to be used in both therapeutic and diagnostic purposes. Similarly, Sun et al. developed an aptamer that was potentially able to prevent the ACE-2 from binding to the spike protein, compete with it, or replace it. Simulated interaction models have also discovered other potential aptamers to bind to the RBD of the spike protein (Song et al., 2020). However, the mutations in the spike protein pose challenges to neutralizing agents that prevent the spike protein and ACE-2 receptors interactions (Li et al., 2020b). To face this challenge, Schmitz et al. introduced an aptamer that was able to bind to the spike protein, but its anti-infection function was regardless of dampening spike and ACE-2 interaction (Schmitz et al., 2021). One possible mechanism behind this finding was claimed to be disrupting the cleavage and conformational changes of the spike protein.

Repurposing the previously introduced aptamers against the SARS-CoV appears to be effective as well (Cho et al., 2011; Parashar et al., 2020). In this respect, aptamers might also interfere with the SARS-CoV-2 replication by targeting the nucleocapsid (N) protein that is highly conserved among coronaviridae families (Parashar et al., 2020; Torabi et al., 2020). Accordingly, Zhang et al. developed DNA aptamers targeting the N protein of SARS-CoV-2 and demonstrated its effectiveness in COVID-19 treatment (Zhang et al., 2020). Targeting the N protein can also be implemented for diagnostic purposes (Chen et al., 2020; Torabi et al., 2020; Zhang et al., 2020). Generally, based on the target, aptamers’ binding ability makes them potential therapeutic and diagnostic agents, both of which possessing great importance in the fight against the COVID-19 pandemic (Liu et al., 2020; Song et al., 2020; Zhang et al., 2020).

Moreover, since nucleic acids are also among the possible targets for aptamers, targeting the conserved domains in the SARS-CoV-2 genome might be on the horizon. In support of this idea, it has been shown that aptamers against highly conserved structural domains of the HIV-1 and HCV RNA genome could inhibit RNA viruses’ replication and particles production *in vitro* by competing with the interactions the domains are involved in or by modifying their structure (Berzal-Herranz and Romero-López, 2020). The study illustrates how aptamers can exploit the structural features of viral genomic RNAs as therapeutic targets.

Compared with other viral-neutralizing agents, while aptamers can have similar high specificity and affinity, they have several advantages over conventional antibodies (Zhou and Rossi, 2017). First of all, the synthesis and selection of aptamers are less time-consuming, less expensive, and can be done entirely *in vitro*. Second, although aptamers lose their folding in inappropriate conditions, they can refold easily by returning the conditions back to normal. In contrast, antibodies denaturation is irreversible which makes them more vulnerable. Third, aptamers can be controllably modified and have faster tissue penetration. In addition, these molecules are less likely to induce a humoral immune response compared to antibodies that induce anti-antibody secretion. Furthermore, they have a broad target range as opposed to antibodies that can target limited antigenic epitopes (Zou et al., 2019).

## siRNAs and Their Therapeutic Potentials Against COVID-19

RNA interference is a natural cell process in which interfering RNA molecules inhibit a gene expression or translation by recognizing and neutralizing its transcript mRNA (Youngren-Ortiz et al., 2016). Among many types of small RNAs that take part in RNA interference-related pathways, siRNAs and micro-RNAs (miRNAs) are the most widely studied and utilized ones (Rosa et al., 2018). Both miRNAs and siRNAs have enormous potentials to be used as therapeutic agents; however, siRNAs have been more considered in clinical applications and drug development experiments because of their higher specificity (Lam et al., 2015). siRNAs use approximately their entire length (typically 21–23 nucleotide) to recognize their target sequence; therefore, they cleave the target mRNA specifically and inhibit a single target gene expression. However, miRNAs mostly use eight nucleotides from their 5′ end to identify targets and have the ability to bind partially complementary sequences; therefore, they can inhibit translation of multiple off-target mRNAs (Chakraborty et al., 2017; Ahmadzada et al., 2018). siRNAs also have some advantages over small molecule drugs or protein therapeutics, such as rapidly designable sequences and relatively simple synthesis (Youngren-Ortiz et al., 2016).

siRNAs are short double-stranded RNAs that consist of a guide (antisense) and a passenger (sense) complementary strands. Once the siRNA enters the cell cytoplasm, it is unwound, and its antisense strand is incorporated within the RNA-induced silencing complex (RISC) and activates it, while the sense strand is degraded. Then, the antisense strand directs the RISC to the complementary target mRNA. mRNA binding to the antisense strand in the activated RISC eventually induces Argonaute endonuclease-mediated mRNA cleavage (Youngren-Ortiz et al., 2016; Ahmadzada et al., 2018).

Over 30 siRNA-based drugs have reached clinical trials for a diverse range of diseases (Hu et al., 2019), and two gained FDA approval among (Le et al., 2020). Patisiran (ONPATTRO^™^) for alleviating the symptoms of the hereditary transthyretin-mediated (hATTR) amyloidosis in adults and givosiran (GIVLAARI^™^) for treating acute hepatic porphyria (AHP) in adults are two siRNA-based drugs from Alnylam therapeutics incorporation that have gained FDA approval (Hu et al., 2019; Kim, 2020). siRNAs also have been being used in clinical trials against hepatitis B and Ebola viral infections in humans and non-human primates, which showed great promise of the siRNA-based therapeutics in virally infected patients (Study of ARB-001467 in Subjects With Chronic HBV Infection Receiving Nucleos(t)Ide Analogue Therapy—Tabular View—ClinicalTrials.gov; Study of ARO-HBV in Normal Adult Volunteers and Patients With Hepatitis B Virus (HBV)—Full Text View—ClinicalTrials.gov; Geisbert et al., 2010; Wooddell et al., 2013; Dunning et al., 2016; Schluep et al., 2017; Saw and Song, 2020).

Several siRNA candidates have been effectively tested against the Severe Acute Respiratory Syndrome Coronavirus (SARS-CoV; Ghosh et al., 2020; Habtemariam et al., 2020; Henzinger et al., 2020; Uludağ et al., 2020). Zheng et al. designed 48 siRNA sequences targeting the entire SARS-CoV genome, among which four siRNAs specific to ORF1b and ORF2 showed potent inhibition of SARS-CoV infection and replication in fetal rhesus kidney (FRhK-4) cells (Zheng et al., 2004). Besides, higher therapeutic activity was observed when a combination of active siRNAs targeting different regions of the viral genome was used. Li et al. showed the efficiency of siRNAs against spike protein-coding and ORF1b (NSP12) regions of the SARS-CoV genome in controlling several SARS symptoms without showing visible toxicity in the rhesus macaque model (Li et al., 2005). Eventually, the study determined low doses of the drug (10–40mg/kg/daily) to be sufficient for satisfactory therapeutic effects in monkeys. siRNAs targeting spike protein-coding regions also have been successfully utilized in other *in vitro* and *in vivo* studies, and they were efficient in low doses and did not show any sign of toxicity (Zhang et al., 2004; Wu et al., 2005; Tang et al., 2008). RdRp, structural envelope (E), membrane (M), and nucleocapsid (N) proteins, as well as 3a and 7a accessory proteins, were also targeted by siRNAs and inhibited the virus replication and production *in vitro* (Lu et al., 2004; Shi et al., 2005; Åkerström et al., 2007; He et al., 2009). Moreover, targeting an essential host protein such as ACE2, as the recognized SARS-CoV receptor, successfully silenced the protein expression in the Vero E6 cells and was influential in reducing the virus infection (Lu et al., 2008). Importantly, implementing multiple siRNAs against different targets or combining siRNAs with other existing antiviral agents showed a synergistic antiviral effect. He et al. reported that targeting multiple structural genes with multiple siRNAs rather than using single siRNA at the same total dosage synergized antiviral effects against SARS-CoV (He et al., 2006, 2009). Besides, it was shown that combining IFN-a with siRNAs against either replicase (R) or membrane 1 (M1) protein reduced viral titers in infected cell line 100 to 1,000 times more than siRNA alone (He et al., 2009).

By around 80% sequence homology with SARS-CoV, expectedly, siRNAs were also quoted as a potential therapy for the SARS-CoV-2 infection (Jamalkhah et al., 2021). Although there are some differences in ORF1a/b, orf8, orf10, and S gene (Kaur et al., 2021), because of high sequence similarity in the replicase domain of ORF1a/b (94.4%), the envelope protein (93.5%) and nucleocapsid protein (88.1%) siRNAs targeting these regions can be utilized for SARS-CoV-2 as well (Lu et al., 2020).

Several *in silico* studies introduced siRNA sequences specific to various genomic sites of the SARS-CoV-2 (Gupta et al., 2020; Patiyal et al., 2020; Chowdhury et al., 2021). In an *in vitro* study, Gallicano et al. showed that siRNAs could inhibit the production of SARS-CoV-2 spike protein in both HEK293 cells and a primary human airway tracheal cell line (Gallicano et al., 2020). They also demonstrated that molecular linking of a cholesterol moiety to the siRNA bypasses the need of prepping the siRNA with any potentially irritating lipid-based transfection reagent in clinical settings. They also showed that the expression of human genomic genes, even those with the highest sequence matches with the siRNA, was not interrupted. Ambike et al. showed that siRNAs targeting viral genomic RNA could terminate viral replication before transcription starts in HEK293T cells (Ambike et al., 2021). In addition to genomic RNA, every sub-genomic RNA containing an identical target sequence was suppressed simultaneously; however, the negative-sense genomic RNA was spared due to inaccessibility for siRNAs. Importantly, the study suggested that targeting sequences that are present in both genomic and sub-genomic RNAs would lower siRNA drug efficacy. The reason proposed to be that highly abundant sub-genomic replicates may compete with the genomic RNA for attaching to siRNAs and/or RISC. Considering that roughly two-thirds of the infected cells’ transcriptome are made up of SARS-CoV-2 RNAs, of which almost all contain the targeted sequences, it seems plausible that targeting sub-genomic RNAs would critically lower the siRNA to gene target ratio (Kim et al., 2020). Accordingly, the study showed that ORF1, which is solely present in genomic RNA, is the most efficient target in the SARS-CoV-2 genome.

In another *in vitro* study, Wu et al. designed 11 siRNAs that target the consensus regions of three key SARS-CoV-2 genes: the spike (S), nucleocapsid (N), and membrane (M) genes. The siRNAs’ effectiveness at silencing viral genes has been determined in human lung and endothelial cells overexpressing the viral genes (Wu and Luo, 2021). Most of the siRNAs significantly decreased the expression of viral genes within 24h, with inhibition rates exceeding 50%. Niktab et al. designed and evaluated the efficacy of six highly specific siRNAs targeting essential viral mRNAs with minimal chance for human genome off-targets. The copy number of viral mRNAs markedly reduced after treatment with the siRNAs *in vitro*; however, *in vivo* inhibition of virus proliferation was unacceptable (Niktab et al., 2021). Interestingly, Ahn et al. utilized miRNA-like off-target activity of siRNAs to inhibit lung fibrosis and collagen producing in human lung cells by adopting seed sequences from antifibrotic miRNAs (Ahn et al., 2021). Accordingly, 13 potential siRNAs whose seed sequences were matched to known antifibrotic miRNAs were tested, and their miRNA-like activity was confirmed. Among them, a siRNA was functionally validated to target the nsp12 region of SARS-CoV-2 encoding RdRP. The siRNA demonstrated similar antifibrotic activity to miR-27a and was experimentally proven to suppress TGF-β-induced lung fibrosis and a collagen-producing gene, COL1A1, in human lung cells. The study suggests that siRNA drugs could potentially inhibit SARS-CoV-2 and attenuate fatal pulmonary fibrosis in COVID-19 at the same time.

siRNA therapy was also applied against SARS-CoV-2 in the *in vivo* models. Idris et al. screened multiple siRNAs targeting highly conserved regions of the SARS-CoV-2 virus and found three candidate siRNAs against viral helicase, RdRp, and 5’UTR that effectively inhibit the virus by greater than 90% either alone or in combination with one another (Idris et al., 2021). Besides, an intravenous liposome delivery platform was applied that resulted in robust delivery of siRNAs to the lungs *in vivo* in the K18-hACE2 mouse model of COVID-19 disease. The study reported marked repression of virus in the lungs and pronounced survival advantage to the treated mice. Khaitov et al. selected a siRNA against the viral RdRp out of 15 in-silico designed siRNAs as the most efficient siRNA inhibiting viral replication *in vitro* (Khaitov et al., 2021). Moreover, they showed that locked nucleic acids (LNAs) modification increases the RNA stability and complexation with the designed peptide dendrimer (KK-46) enhances cellular uptake to allow topical application by inhalation of the final formulation. A significant reduction of virus titer and lung inflammation in animals exposed to inhalation of the siRNA was shown.

## Aptamer–siRNA Chimera Against SARS-CoV-2

Since aptamers and siRNAs are both nucleic acids, conjugating them to produce aptamer–siRNA chimeras (AsiCs) is possible either through covalent linkage or complementation (Kruspe and Giangrande, 2017). Aptamer-mediated siRNA delivery was first described by two independent research groups in 2006. Both groups used RNA aptamers targeting prostate-specific membrane antigen (PSMA) to deliver siRNAs to prostate cancer cells specifically (Kruspe and Giangrande, 2017; Soldevilla et al., 2018). Since then, many similar studies were performed to target different antigens from many cancer types such as breast, glioblastoma, T cell lymphoma, and melanoma (Soldevilla et al., 2018). These studies corroborated the potentials of aptamers as excellent candidates for siRNA delivery because of their low toxicity and high affinity and specificity to targets (Zou et al., 2019). Beyond siRNA delivery, AsiCs can be used as dual functioning agents that proceed both aptamer-mediated receptor suppression/activation in parallel with siRNA-mediated gene interference (Kruspe and Giangrande, 2017). For instance, 4-1BB aptamer–siRNA conjugate was used to deliver a siRNA against mTOR complex 1 (mTORC1) into CD8+ T-cells concurrent with the 4-1BB T-cell stimulatory receptor activation (Berezhnoy et al., 2014).

Besides cancer therapy, antiviral therapy is an area in which AsiCs have been widely tested as both targeted siRNA delivery strategy and dual-functioning virus-neutralizing and replication-interfering agents (Takahashi et al., 2015). Most AsiCs designed and tested to treat viral infections were directed against HIV. The HIV trans-activator (tat) and the regulator of expression of virion proteins (rev) are the two essential regulatory elements of HIV, where their coding regions in the 9.2kb RNA genome of the virus have been widely targeted *via* siRNAs. These siRNAs have been successfully conjugated with RNA-aptamers that bind to the HIV-1 protein gp120, and CD4 and CCR5 on the T cells surface to form anti-HIV aptamer–siRNA chimeras (Kruspe and Giangrande, 2017).

Cell-surface CD4 and CCR5, which are required for HIV-1 entry to cells, were targeted by AsiCs with the rationale of delivering anti-HIV siRNAs or preventing *de novo* infection of uninfected CD4+ cells (e.g., CD4+ T cells, macrophages; Takahashi et al., 2015). As a targeted siRNA delivery tool, a DNA aptamer was obtained by converting a reported RNA aptamer that binds to CD4 protein. The aptamer was conjugated with a siRNA targeting HIV-1 protease. The resulting DNA aptamer–siRNA chimera was able to specifically enter into CD4+ T cells and efficiently knockdown the expression of the exogenous HIV protease gene (Zhu et al., 2012). Interestingly, wheeler et al. proved that AsiCs could be used as prophylactic agents for HIV infection (Wheeler et al., 2011, 2013). They showed that the CD4 aptamer–siRNA chimeras efficiently suppressed viral gene expression in CD4+ T cells and macrophages *in vitro*, in polarized cervicovaginal tissue explants, and the genital tract of humanized mice. This suppression led to protection against HIV vaginal transmission. However, while the CD4 aptamer alone inhibited HIV transmission, CD4 aptamer–siRNA chimeras demonstrated more significant inhibitory effects than the aptamer itself. Applying the CD4 aptamer–siRNA to humanized mice resulted in efficient protection against HIV-1 infection with no detectable viral load up to 12weeks in chimera treated mice group. Another study has shown that the CCR5 aptamer-TNPO3 chimera leads to more robust and more prolonged suppressions against HIV-1 replication in human peripheral mononuclear cells than the CCR5 targeting aptamer alone (Zhou et al., 2015).

The first gp120 aptamer–siRNA chimera was created by combining a neutralizing aptamer against gp120 with siRNA targeting the HIV-1 tat/rev common exon sequence (Zhou et al., 2008). The study showed both aptamers and chimeras suppress HIV-1 replication and production dramatically. Anti-gp120 aptamer bound directly to HIV virions or intracellular gp120 and was an effective delivery vector for siRNAs targeting viral replication. AsiCs were also studied *in vivo* in 2011, which showed that the gp120 aptamer–tat/rev siRNA chimera suppressed the viral load within a week from the last injection, and the suppression persisted throughout the treatment period without showing any immune response reaction. Furthermore, chimera treatments prevented HIV-1-induced depletion of helper CD4+ T cells, a significant characteristic of HIV-1 infection at the acute stage (Neff et al., 2011). Similar results were also obtained in other studies employing gp120 aptamer–tat/rev siRNA chimera in various constructs (Zhou et al., 2009, 2013, 2018).

Altogether, it can be concluded that AsiCs can be a rational anti-SARS-CoV-2 drug, and at least they can be tested against coronaviruses. The viral genome can be more efficiently targeted using RNA-aptamers that bind to the SARS-CoV-2 protein S or ACE2 receptor on the lung cells’ surface. Such AsiCs would represent both viral neutralization and RNA interference in one structure without showing considerable immune response. Considering that SARS-CoV-2 is evolving and multiple strains with their specific mutation patterns coexist, combining multiple drugs acting through various mechanisms has proven to minimize resistant mutation appearance and improve the effectiveness (Zeng et al., 2021). Apart from possible dual targeting, AsiCs can mediate a targeted delivery of siRNAs to lung cells which minimize the possible off-target harms in using anti-COVID siRNAs. Using such drugs to reduce the viral load transiently for several weeks could be vital for COVID-19 patients and can buy time for the immune system to limit the infection itself, which is not expected in HIV infection.

### Challenges of Nucleic Acid-Based Therapies and Possible Solutions

Nucleic acid-based therapies have surely great potential in COVID-19 treatment. However, there are still many challenges that need to be resolved. Several issues have been reported when developing RNA-based vaccines for COVID-19, to which RNA-based therapies can also be subjected. The first and most prominent challenge in RNA application is *in vivo* instability. Unmodified RNA molecules are highly prone to degradation by nucleases that prevail in extracellular space, and negatively charged RNA molecules cannot readily pass through the hydrophobic cytoplasmic membrane, limiting their pharmacokinetics. Particularly, the low molecular weight and diameter of aptamers and siRNAs ease their filtration from kidneys and hence interfere with their therapeutic effect in the body (Burgess, 2012; Nimjee et al., 2017; Lundstrom, 2020; Kulkarni et al., 2021).

A handful of strategies including chemical modification, more stringent purification methods, and employment of suitable delivery systems have been exploited to address the mentioned pharmacokinetic challenges. Like COVID-19 mRNA vaccines, that harbor multiple chemically modified nucleotides and poly-Adenine tails, aptamers and siRNAs can benefit from a handful of modifications as well. For example, PEG-conjugated aptamers have higher bioavailability and circulating half-life *in vivo*. Besides, protective functional group incorporation (e.g., thiol-phosphate, 2′-Fluoro, 2′-amino, etc.) in the phosphate backbone or 2′-position of the ribose sugar can improve nuclease resistance and binding affinity (Kuwahara and Sugimoto, 2010; Zhou et al., 2015; Kimoto et al., 2016; Hirao et al., 2018; Chernikov et al., 2019). Similarly, chemical modifications of siRNAs have been proven to be crucial for pharmacokinetics and pharmacodynamics enhancement while preserving target affinity and efficacy. Lipid nanoparticle (LNP) encapsulation is another strategy that is widely used to mediate the preservation of the negatively charged RNA molecules, particularly COVID-19 mRNA vaccines, from degradation and their passage across the cellular membrane (Liu et al., 2021). Similar to RNA vaccines, local or topical delivery of therapeutic siRNAs and implementing diverse types of nanocarriers resulted in much higher cellular uptake (Kumar et al., 2014). Also, to enhance siRNA uptake by the cells of interest, siRNAs could also be targeted by directly conjugating them to antibodies, single-chain variable fragments, aptamers, and receptor ligands (Hu et al., 2019). GalNAc-siRNA conjugate is an example of targeting siRNAs to liver cells, and one GalNAc-siRNA conjugate drug, givosiran (GIVLAARI^™^), obtained FDA approval for treating AHP in adults (Brown et al., 2021).

Aberrant immune stimulation and immunogenicity is another area of concern for both RNA-based vaccines and therapeutics. Contamination that may remain after *in vitro* synthesis and LNP delivery systems in mRNA vaccines can lead to local and systemic inflammatory and immunogenic responses which are possibly accompanied by autoreactive antibodies (Piyush et al., 2020). Likewise, although there is limited information, there are still concerns about the toxicity and immunogenicity of aptamers due to inadvertent accumulation and non-specific effects *in vivo* (Zhou and Rossi, 2017). siRNAs have also been reported to pose aberrant innate immunity induction either by siRNA or its delivery vehicles (Chakraborty et al., 2017; Le et al., 2020). Toll-like receptor-mediated recognition of siRNAs can trigger innate immune stimulation. As an attempt to abolish this aberrant immune-reactivity, non-immunostimulatory siRNAs have been introduced that carry 2′-O-ribose methylation. This RNA modification eliminates the unwanted immunostimulatory capacity of siRNAs (Hamm et al., 2010). Higher purity of RNA therapeutics reduces the chance of unwanted immune reactions. Thus, to eliminate impurities within the *in vitro* transcribed RNA, the high liquid chromatography purification method is utilized which mediates the removal of double-stranded RNA contaminants, which in turn decreases the production of type 1 interferon and pro-inflammatory cytokines (Karikó et al., 2011).

In addition, siRNAs may affect other transcripts sharing limited complementarity to the RNA duplex, displaying unintentional off-target silencing. A particular position-specific chemical modification of siRNA, 2′-O-methyl ribosyl substitution at position 2 in the guide strand has therefore been developed that can substantially revert the silencing of partially complementary transcripts (Jackson et al., 2006).

## Conclusion

Here, literature was reviewed to assess the plausibility of aptamers, siRNAs, and their conjugate as potential drugs against the SARS-CoV-2. Ongoing mutations that take place in the SARS-CoV-2 genome may have strong influences on the host range, tissue tropism, and pathogenicity of the virus, as well as the response to drugs and vaccines. Therefore, the fact that aptamers, siRNAs, and aptamer–siRNA chimeras can be altered easily to accommodate the virus’s genetic mutations makes them an attractive approach for anti-SARS-CoV-2 therapy. Moreover, former successful applications of these molecules against viruses, besides their major advantages such as low toxicity, high specificity, and relatively simple synthesis with low costs, might provide sufficient rationale for applying these nucleic acid-based drugs against the SARS-CoV-2. Such application would require precise modifications of the structures to overcome the barriers that hindered the vast utilization of the drugs in the clinical settings, and future studies are warranted to evaluate their potential efficacy and safety.

## Author Contributions

JK, MA-K, YA, and MJ searched in the literature and wrote the manuscript. JK supervised the group and revised the manuscript. All authors read and approved the final version of the manuscript.

## Conflict of Interest

The authors declare that the research was conducted in the absence of any commercial or financial relationships that could be construed as a potential conflict of interest.

## Publisher’s Note

All claims expressed in this article are solely those of the authors and do not necessarily represent those of their affiliated organizations, or those of the publisher, the editors and the reviewers. Any product that may be evaluated in this article, or claim that may be made by its manufacturer, is not guaranteed or endorsed by the publisher.

## Abbreviations

COVID-19: Coronavirus Disease 2019
SARS-CoV-2: Severe Acute Respiratory Syndrome Coronavirus 2
SELEX: Systematic Evolution of Ligands by Exponential Enrichment
siRNA: Small interfering RNA
miRNA: microRNA
RISC: RNA-induced silencing complex
AsiCs: Aptamer–siRNA chimeras
gp-120: Glycoprotein 120
HIV: Human immunodeficiency virus
RBD: Receptor-binding domain
